# Oral health status and oral habits of children and adolescents with hemophilia: a report from the children’s hemophilia comprehensive care center of China

**DOI:** 10.1007/s00431-023-05270-z

**Published:** 2023-11-07

**Authors:** Yue Li, Guoqing Liu, Runhui Wu, Guoxia Yu

**Affiliations:** 1 Department of Stomatology, Beijing Children’s Hospital, Capital Medical University, National Center for Children’s Health, Nanlishi Road 56, Xicheng District, Beijing, 100045 China; 2 Department of Hematology, Beijing Children’s Hospital, Capital Medical University, National Center for Children’s Health, Beijing, 100045 China

**Keywords:** Hemophilia, Children, Oral health, Caries, Oral hygiene

## Abstract

In recent years, the diagnosis and treatment of hemophilic children in China has significantly improved. However, oral health conditions, which affect quality of life, haven’t received attention in this population. To explore the oral health status and oral hygiene of children and adolescents with hemophilia in the Children’s Hemophilia Comprehensive Care Center of China. Dental and oral hygiene examinations were performed in children and adolescents with hemophilia who visited Beijing Children’s Hospital. DMFT/dmft (decayed, missing, filled teeth in permanent and primary teeth) was assessed according to World Health Organization (WHO) criteria. The simplified oral hygiene index (OHI-S) was used to evaluate the oral hygiene condition of the subjects. Questionnaires were completed by their parents. SPSS 21.0 was used for statistical analysis. A total of 114 children and adolescents were enrolled. The caries prevalence was 57.4%, 72.2% and 41.2% in primary, mixed and permanent dentitions respectively. The filling rates were 14.4%, 13.9%, and 11.4%, respectively, and the OHI-S scores of the three dentition groups were 1.49 ± 0.46, 1.57 ± 0.43, and 1.76 ± 0.46, respectively. A total of 103 valid questionnaires were collected. Sixty-nine children (67%) didn’t brushed their teeth 2 times a day. Nearly half of the parents knew little about fluoride toothpaste. Multiple linear regression analysis revealed that brushing teeth with the help of parents had a significant positive impact on OHI-S.

*  Conclusion*: Dental health was unsatisfactory among hemophilic children and adolescents. The caries filling rates were low. Patients and their parents did not give much attention to oral health.

**Table:** 

**What is Known:** *• Caries and gingivitis are the two main oral diseases that affect children with hemophilia.* *• However, the oral health conditions of children and adolescents with hemophilia have not received much attention in China.*	
**What is New:** *• This is the first study concentrating on the dental health of children with hemophilia in China.* *• Dental health was unsatisfactory among children and adolescents with hemophilia in China.*

**What is Known:**

*• Caries and gingivitis are the two main oral diseases that affect children with hemophilia.*

*• However, the oral health conditions of children and adolescents with hemophilia have not received much attention in China.*

**What is New:**

*• This is the first study concentrating on the dental health of children with hemophilia in China.*

*• Dental health was unsatisfactory among children and adolescents with hemophilia in China.*

## Introduction

Hemophilia is a hereditary hemorrhagic disease caused by congenital coagulation factor deficiency. It is classified as A and B based on the deficiency of clotting factors VIII and IX [1]. The severity is classified based on the residual activity of the defective factor as mild (5–40%), moderate (1–5%) and severe (< 1%) [2]. The main clinical manifestations are spontaneous or post injury bleeding in joints, muscles and other parts. Oral bleeding is common in patients with hemophilia, due to poor oral hygiene, traumatic injuries and eruption and exfoliation of teeth [3].

Epidemiological investigation shows that the incidence of hemophilia in China ranges from 2.73 to 3.09 per 100,000 [4]. From 2007 to 2019, a total of 17,779 patients with hemophilia A were identified in the Haemophilia Treatment Centre Collaboration Network of China (HTCCNC) database across 31 divisions in China [5]. From 2007 to 2021, 3782 patients with a confirmed diagnosis of hemophilia B were identified in the HTCCNC database, among them 43.5% were children under 18 years old [6]. In recent years, although the diagnosis and treatment of hemophilia in children in China has significantly improved, there are still cases of untimely diagnosis and inadequate treatment [7]. Because it is a rare disease, most dentists in China have little experience in dealing with children with hemophilia. Similar to the average healthy children population, the two main oral diseases affecting children with hemophilia are caries and gingivitis. The oral health of children with hemophilia has been studied in many countries. Comparisons of oral status between patients with hemophilia and healthy controls were conducted. A higher prevalence of caries has been found in hemophilia patients than in healthy controls in several studies [8, 9]. However, some studies noted lower caries experiences among children with hemophilia [10–12]. Zaliuniene et al. [13] found that in children with hemophilia their deciduous teeth had a lower caries experience, while in older children or adults with permanent dentitions, there was no significant difference between cases and controls.

In China, the oral hygiene of adult patients and healthy groups has been compared, and the results showed that patients with hemophilia had poor oral health, and their awareness of oral health was lacking [14, 15]. However, the oral health condition of children with hemophilia in China has not been studied. The reasons may be that hemophilia patients in China rarely pay much attention to oral health, and the number of patients seeking dental treatment is limited. Therefore, data on the oral health of this population is currently limited. The aim of this study was to investigate oral health status and oral habits in children and adolescents with hemophilia referred to the Children’s Hemophilia Comprehensive Care Center of China.

## Materials and methods

From April to August 2022, children and adolescents with hemophilia A and hemophilia B seen at the outpatient clinic of hematology in Beijing Children's Hospital, which is the Children’s Hemophilia Comprehensive Care Center of China, were enrolled. Exclusion criteria were as follows: any other coagulation disorders, systemic disease, uncooperative patients, and refusal to participate in the study. The study design, protocol, and informed consent were approved by the Ethics Committee of Beijing Children’s Hospital, Capital Medical University ([2022]-E-159-Y). And written informed consent was obtained from the parents of all children enrolled in this study.

The patients were divided into three groups: the primary dentition group, mixed dentition group and permanent dentition group. Dental examination and oral hygiene examination of these children were conducted. For primary teeth, decayed, missing and filled teeth (dmft) and decayed, missing and filled surface (dmfs) indices were recorded. Decayed, Missing and Filled Teeth (DMFT) and Decayed, Missing and Filled Surface (DMFS) indices were recorded for permanent teeth, according to the World Health Organization (WHO) criteria [10].

The simplified oral hygiene index (OHI-S) was used to evaluate the oral hygiene of the subjects [16]. It consisted of the debris index-simplified (DI-S) and calculus index-simplified (CI-S). For primary teeth, the plaque/calculus of the labial surface of primary maxillary right central incisor and primary mandibular left central incisor, buccal surface of primary maxillary right second molar and primary maxillary left second molar, and lingual surface of primary mandibular left second molar and primary mandibular right second molar were examined. If the primary second molars had not yet erupted, the examined teeth were replaced with the primary first molar. For permanent teeth, the plaque/calculus of the labial surface of permanent maxillary right central incisor and permanent mandibular left central incisor, buccal surface of permanent maxillary right first molar and permanent maxillary left first molar, and lingual surface of teeth permanent mandibular right first molar and permanent mandibular left first molar were checked. The score was recorded according to the plaque/calculus area of the tooth surface. Scores of 0, 1, 2, and 3 were scored as no plaque/calculus, plaque/calculus area < 1/3, plaque/calculus area < 2/3, and plaque/calculus area > 2/3, respectively. Only fully erupted teeth were scored. Natural teeth with full crown restorations and surfaces reduced in height by caries or trauma were not scored. The calculation of DI/CI = total score/ number of surfaces examined. The calculation of OHI-S = DI-S + CI-S [17].

The questionnaire was distributed and completed by parents. Questions on eating habits, oral hygiene habits, oral hemorrhage and dental experiences were included. Eating habits refer to the habit of eating sweets and beverages. Oral hygiene habits refer to the habit of brushing teeth. The questions in the questionnaire refer to the 4^th^ Nation Oral Health Epidemiological Survey of China [18] and literature on oral investigation in children with hemophilia [19].

## Statistical analysis

Descriptive statistics were conducted on the demographics of the children enrolled in this study. dt/DT, dmft/DMFT, OHI-S of each dentition group were calculated and expressed as means (and standard deviations). The OHI-S of different dentition groups was compared using one-way ANOVA. And descriptive analysis was conducted on the results of the questionnaire. Multiple linear regression analysis was used to explore the association between oral habits with oral health status. Statistical significance was defined as p < 0.05. Data analyses were performed using SPSS 21.0.

## Result

A total of 114 patients were enrolled in this study. The children were all male, with a minimum age of 1 year and a maximum age of 15 years. The median age was 5.5 years. Among them, 100 had hemophilia A, and 14 had hemophilia B. Regarding severity, 3 were mild, 9 were moderate and 102 were severe. Sixty-one children had primary dentition, 36 had mixed dentition, and 17 had permanent dentition.

In different dentition groups, caries rate, dt (decayed teeth), dmft (decayed/ missing/ filling teeth), filling rate, and OHI-S score were calculated (Table 1).

**Table 1:** Decayed, missing, filled teeth and OHI-S in primary, mixed, permanent dentition

	Caries prevalence	dt/DT (mean ± SD)	dmft/DMFT (mean ± SD)	Filling rate	OHI-S
Primary dentition	57.4%(35/61)	3.41 ± 4.24	3.85 ± 4.64	14.4%(30/208)	1.49 ± 0.46
Mixed dentition	72.2%(26/36)	3.64 ± 3.46	4.25 ± 3.64	13.9%(17/122)	1.57 ± 0.43
Permanent dentition	41.2%(7/17)	2.06 ± 3.03	2.2 ± 3.33	11.4%(4/35)	1.76 ± 0.46

In the primary dentition group, caries were found in 35 children (57.4%). A total of 208 teeth were decayed. Fillings were detected in 30 teeth of 7 children, and only 1 child had all his caries treated. The mean dmft of ages 1 to 5 were 0.6, 1.27, 4.22, 5.36, and 7.09, respectively. Both the incidence and prevalence of caries in primary teeth increased with age.

In the mixed dentition group, caries were found in 26 children (72.2%). Fillings were detected in 9 children, among which 2 had all their caries teeth treated.

In the permanent dentition group, caries were found in 7 patients (41.2%). Fillings were detected in only 1 patient.

The OHI-S scores of 3 groups are shown in Table 1. The OHI-S score is classified as excellent (score 0), good (scores from 0.1–1.2), fair (scores from 1.3–3.0), and poor (scores from 3.1–6) [17]. According to these criteria, the OHI-S scores of the three groups were all judged to be fair. One-way ANOVA indicated no significant difference between the three groups (*p* = 0.24).

A total of 103 valid questionnaires were collected. Eleven were excluded due to inconsistent answers in their questionnaires. Fifty-five children were in primary dentition. Thirty-four were in mixed dentition. Fourteen were in permanent dentition. Eating habits and oral hygiene habits of different dentition groups were listed in Table 2. Sixty-nine children out of 103 (67%) didn’t brushed their teeth 2 times a day. Nearly half of the parents knew little about fluoride toothpaste.

**Table 2:** Eating habits and oral hygiene habits of primary, mixed, permanent dentition

	Primary dentition	Mixed dentition	Permanent dentition	Total
Frequency of eating sweets every day				
More than 3 times a day	7(12.7%)	4(11.8%)	2(14.3%)	13(12.6%)
1-2times a day	29(52.7%)	18(52.9%)	6(42.85%)	53(51.5%)
occasionally	19(34.6%)	12(35.3%)	6(42.85%)	37(35.9%)
Sweets or beverages before bed				
Frequently	4(7.3%)	3(8.8%)	0	7(6.8%)
occasionally	26(47.3%)	14(41.2%)	6(42.9%)	46(44.7%)
never	25(45.4%)	17(50%)	8(57.1%)	50(48.5%)
Frequency of brushing teeth				
2 or more times a day	12(21.8%)	19(55.9%)	3(21.4%)	34(33%)
1 time a day	26(47.3%)	14(41.2%)	10(71.4%)	50(48.5%)
occasionally/never	17(30.9%)	1(2.9%)	1(7.2%)	19(18.5%)
Brushing teeth with the help of parents				
2 or more times a day	7(12.7%)	2(5.9%)	0	9(8.7%)
1 time a day	20(36.4%)	2(5.9%)	0	22(21.4%)
occasionally/never	28(50.9%)	30(88.2%)	14(100%)	72(69.9%)
Fluoride toothpaste				
Yes	21(38.2%)	14(41.2%)	3(21.4%)	38(36.9%)
No	10(18.2%)	2(5.9%)	2(14.3%)	14(13.6%)
Don’t know	24(43.6%)	18(52.9%)	9(64.3%)	51(49.5%)

Multiple linear regression analysis was used to explore the association between oral habits with oral health status. The results suggested that dt/DT, dmft/DMFT, and OHI-S were not related to the frequency of eating sweets, sweets or beverages before bed, the frequency of brushing teeth, and whether to use fluoride toothpaste. Brushing teeth with the help of parents had a significant positive impact on OHI-S, but was not related to dt/DT/dmft/DMFT.

We also surveyed dental visits and oral bleeding in the questionnaire. 

Ten children with primary dentition (18.5%), 21 children with mixed dentition (61.8%) and 8 children with permanent dentition (57.1%) had visited dentists in the past.

Fifteen children with primary dentition (27.8%), 18 children with mixed dentition (52.9%) and 9 children with permanent dentition (64.3%) experienced oral hemorrhage. The two most common causes of oral bleeding were trauma (11/15 in primary dentition, 7/18 in mixed dentition, 3/9 in permanent dentition) and replacement of teeth (10/18 in mixed dentition, 2/9 in permanent dentition). The other reasons include spontaneous gingival bleeding, gingival bleeding when brushing teeth and viral infection.

## Discussion

In recent years, low-dose prophylaxis treatment has been shown to be effective and widely used in China [20]. Moreover, children’s hemophilia treatment centers and comprehensive care teams have been constantly establishing and improving. Along with systemic health care, dental care is also important for children with hemophilia. However, there is little information on the oral health status of these children in China. Thus, the present study investigated the dental health of children with hemophilia who visited Beijing Children’s Hospital, which is the Children’s Hemophilia Comprehensive Care Center of China.

This is the first study concentrating on the dental health of hemophilic children in China. The oral health of children is more likely to be neglected in China. According to the 4^th^ Nation Oral Health Epidemiological Survey in China, the caries rates of primary teeth in 3-, 4-, and 5-year-old groups were 50.8%, 63.6%, and 71.9%, respectively. However, the filling rates were only 1.5%, 2.9%, and 4.1%, respectively. The caries rates of permanent teeth in 12- and 15-year olds were 38.5% and 44.4%, respectively. The filling rates were only 16.6% and 18.5%, respectively [18].

In this study, we obtained similar results in children with hemophilia. The prevalence of caries was high, but filling rates were relatively low. The caries rate in primary dentition was 57.4%, and in the 3-, 4-, and 5-year-old groups, it was 61.6%, 81.8%, 63.6%, respectively. In permanent dentition, the caries rate was 41.2%. The filling rates were low, namely, 14.4% in primary dentition, 13.9% in mixed dentition and 11.4% in permanent dentition. We can see a worse tendency in children with hemophilia. However, no statistically significant difference was found in dmft in primary dentition and DMFT in permanent dentition between children with hemophilia and normal children. The reason may be that the sample size was limited, and we only collected data from children with hemophilia who came to Beijing Children’s Hospital.

The OHI-S was improved to be an effective method for assessing oral hygiene in population groups and has been widely used [16]. The OHI-S score is classified as excellent (score 0), good (scores from 0.1–1.2), fair (scores from 1.3–3.0), and poor (scores from 3.1–6) [17]. Most of the children recruited in our study had plaque accumulation. Only 1 child in permanent dentition showed calculus accumulation in checked teeth surfaces. In our study, the three groups were all judged to be fair (1.3–3.0). A previous study found worse oral hygiene in adults with hemophilia than in healthy controls in China [15]. A study conducted in Kuala Lumpur found a low OHI-S in the hemophilic group [21]. There were no relevant data on healthy children and adolescents in China. According to 4^th^ Nation Oral Health Epidemiological Survey, the periodontal health rates in the 12-year-old group and 15-year-old group were only 41.6% and 34.8%, respectively [18]. Whether the oral hygiene of children with hemophilia is worse than that of normal children in China needs further study.

Oral health awareness of hemophilia in China needs improvement. Li Quixing et al. found poor oral health awareness and behavior in adults with hemophilia [14]. The results of the questionnaire showed that many children had not effectively brushed their teeth. Only 7 children in primary dentition and 2 children in mixed dentition had their teeth brushed by their parents 2 times a day. Parents of 24 children in primary dentition, 18 children in mixed dentition, and 9 children in permanent dentition had no idea of the caries prevention effect of fluoride toothpaste. Only 39 children (34.2%) in this study had visited dentists in the past, but few visited dentists regularly. Most parents and children focused mostly on the disease hemophilia and gave most of their attention to avoid bleeding. Oral health was often neglected. There is still a long way to go in the oral health education of parents and children with hemophilia.

There existed some limitations in our study. There may be recall bias when parents filling the questionnaire. And this study only investigated children with hemophilia who came to Beijing Children’s Hospital. The results may not represent all regions of China. A multicenter study in China will be considered in the future to verify the results.

## Conclusion

Dental health and oral hygiene were unsatisfactory in children with hemophilia in Beijing Children’s Hospital. The caries prevalence rates were high, and filling rates were low. Patients and their parents did not give much attention to oral health.

## Data Availability

The data in the current study are available from the corresponding author on reasonable request.

## Abbreviations

CI-S: Calculus index-simplified
DI-S: Debris index-simplified
DMFT/dmft: Decayed, missing, filled teeth in permanent and primary teeth
DMFS/dmfs: Decayed, missing, filled teeth in permanent and primary teeth surface
dt: Decayed teeth
OHI-S: Simplified oral hygiene index
WHO: World Health Organization
